# Stage IA and IC adult granulosa cell tumors: Clinical features, long-term outcomes and prognostic factors in a 333-patient cohort over three decades

**DOI:** 10.1007/s10147-025-02897-w

**Published:** 2025-10-14

**Authors:** Jingwen Gan, Xiao Ma, Ying Cao, Dongyan Cao, Huimei Zhou, Mei Yu, Tao Wang

**Affiliations:** National Clinical Research Center for Obstetric & Gynecologic Diseases, Department of Obstetrics and Gynecology, Peking Union Medical College Hospital, Chinese Academy of Medical Sciences & Peking Union Medical College, Peking Union Medical College Hospital (Dongdan Campus), No.1 Shuaifuyuan Wangfujing Dongcheng District, Beijing, 100730 China

**Keywords:** Adult granulosa cell tumor, Early-stage, Fertility-sparing surgery, Surgical staging, Adjuvant chemotherapy, Disease-free survival

## Abstract

**Background:**

Adult granulosa cell tumors (AGCTs) are rare low-grade malignant ovarian tumors, with 80–90% diagnosed at FIGO stage I. This study aimed to identify prognostic factors and refine management for stage I AGCT.

**Methods:**

In this 30-year retrospective cohort study, patients with stage I AGCT between January 1988 and January 2024 were selected and reviewed in total cohort and subgroups according to tumor stage.

**Results:**

This retrospective study analyzed 333 eligible AGCT cases, including 196 patients (58.9%) with FIGO stage IA and 137 (41.1%) with stage IC. After a median follow-up of 138.5 ± 108.0 months, recurrence occurred in 55 patients (40.1%) in the IC group, significantly higher than that in the IA group (38 patients, 19.4%; *P* < 0.001, FDR = 0.005). Approximately half of recurrences in both groups were intra-abdominal, with comparable median recurrence intervals. Multivariate logistic regression identified stage IC (*P* = 0.001), incomplete staging surgery (*P* = 0.015) and adjuvant chemotherapy (*P* = 0.002) independent predictors of increased recurrence. In the propensity-matched cohort (*n* = 188), adjuvant chemotherapy showed no significant association with recurrence (*P* = 0.067). Cox multivariate analysis revealed stage IC and incomplete staging surgery as independent prognostic factors for worse DFS in early-stage disease (*P* = 0.001 and 0.012, respectively). Notably, complete staging surgery was associated with improved DFS specifically in stage IC patients (*P* = 0.016).

**Conclusions:**

Unilateral salpingo-oophorectomy instead of simple cystectomy demonstrated a favorable safety profile in reproductive patients with stage I AGCT. Comprehensive surgical staging without lymphadenectomy should be considered as a viable treatment strategy, especially in the stage IC disease, manifesting a significantly higher recurrence rate and shorter DFS compared to stage IA counterparts.

**Supplementary Information:**

The online version contains supplementary material available at 10.1007/s10147-025-02897-w.

## Introduction

Ovarian adult granulosa cell tumors (AGCTs), a rare subtype of sex cord-stromal tumors, account for approximately 5% of all ovarian malignancies [1, 2]. Characterized by their indolent growth pattern and endocrine activity, AGCTs are typically diagnosed at an early stage due to symptoms related to hormone secretion, such as abnormal uterine bleeding or palpable pelvic masses [3]. Despite favorable long-term survival rates in stage I patients, late recurrences—occurring even decades after initial diagnosis—remain a significant clinical concern, with reported recurrence rates ranging from 10 to 30% [4, 5].

Current management of stage I AGCT relies primarily on surgical resection, yet notable uncertainties persist regarding the extent of surgical procedure, adjuvant treatment strategies and risk stratification [6–8]. Existing studies on prognostic factors, such as patient’s age, tumor size, tumor rupture and histological patterns, have yielded conflicting results [8, 9], partly due to the rarity of the disease and limited long-term follow-up data. The identification of reliable and more accurate histopathological characteristics of AGCT is crucial to assist gynecologic oncologists in predicting which individuals are at higher risk of recurrence, thereby facilitating the development of optimized management recommendations for AGCT.

The purpose of our study was to analyze clinical and histopathological parameters of FIGO stage I AGCT patients and evaluated their impact on recurrence, disease-free survival (DFS), and overall survival (OS). Since stage IC tumor had reportedly higher recurrence rates (43%) compared to those with stage IA disease (24%) [10], we also evaluated the key prognostic factors in both subgroups, aiming to refine risk-adapted surveillance strategies and guide personalized therapeutic decisions for this unique patient population.

## Method

### Study design and patient selection

This retrospective observational study analyzed clinical data from 333 consecutive patients diagnosed with International Federation of Gynecology and Obstetrics (FIGO) stage I adult-type granulosa cell tumor (AGCT) at Peking Union Medical College Hospital between January 1988 and January 2024. Exclusion criteria included: 1) loss to follow-up within 6 months post-surgery; 2) concurrent malignancies (except endometrial carcinoma); 3) prior history of radiotherapy or chemotherapy. Based on FIGO staging criteria, the cohort was stratified into stage IA (n = 196) and stage IC (n = 137) subgroups. These patients were followed up until December 2024 or until death, with particular focus on clinicopathological predictors of tumor recurrence and DFS. The study protocol was approved by the Institutional Review Board of Peking Union Medical College Hospital (Approval No. I-24PJ430) with waiver of informed consent due to the retrospective nature.

### Data collection

A comprehensive dataset of demographic and clinical parameters was retrospectively collected, including patient age, presenting symptoms, preoperative serum cancer antigen 125 (CA125) level, maximum tumor diameter, FIGO stage, surgical approach, adjuvant treatment, time to recurrence, and survival status at last follow-up. Longitudinal follow-up data were obtained through structured outpatient medical records supplemented by standardized telephone interviews.

OS was calculated from the date of histological diagnosis to death from any cause. DFS was defined as the interval between primary treatment completion and the first radiologically confirmed recurrence. Fertility-sparing surgery (FSS) aimed to preserve the anatomical integrity of at least one ovary with its fallopian tube and the uterus. Radical surgery (RS) comprised hysterectomy with bilateral salpingo-oophorectomy as the baseline procedure. Complete surgical staging included abdominopelvic cavity assessment, complete omentectomy or omental biopsies, and systematic peritoneal sampling. Staging adhered to the FIGO 2014 criteria for ovarian cancer. Cases that did not undergo any component of this staging procedure during initial or secondary surgery were classified as incomplete staging. For patients with high- or intermediate-risk FIGO stage I disease (large tumor size, intraoperative tumor rupture, stage IC, poor differentiation), senior surgeons determined the need for concurrent lymphadenectomy and postoperative platinum-based chemotherapy. Although chemotherapeutic regimens evolved during the 36-year study period (e.g., increased use of paclitaxel -carboplatin after 2005), indications for adjuvant therapy—stage IC, tumor rupture, large size, or poor differentiation—remained consistent. In cases with confirmed metastatic disease, cytoreductive surgery aimed to achieve complete macroscopic resection.

### Follow-up

Following completion of primary therapy, patients underwent a structured surveillance protocol: quarterly follow-up evaluations for the initial two years, semiannual assessments over the subsequent three years, and annual monitoring thereafter. The surveillance regimen comprised systematic clinical examinations, serial serum tumor marker analyses (including CA125 and anti-Müllerian hormone), and abdominopelvic imaging via ultrasound or contrast-enhanced computed tomography (CT). Disease recurrence was defined radiologically by the identification of new measurable lesions (≥ 10 mm in maximal diameter) on follow-up imaging. Elevated serum markers (inhibin B and AMH) provided supportive evidence. Although histological examination remains the diagnostic gold standard, it was not routinely required for confirmation in this context, as early-stage recurrences often lack an indication for immediate biopsy or surgery.

### Statistical analysis

All analyses were conducted using SPSS software (version 22.0; Chicago, IL, USA). Missing data (tumor size: 19.8%; CA125: 15.0%) were addressed via multiple imputation, generating five datasets using regression models with age, FIGO stage, recurrence status, and surgical approach as predictors. Pooled estimates from imputed datasets were utilized in final analyses.

Continuous variables were expressed as mean ± standard deviation (normally distributed) or median (range) (non-normally distributed). Intergroup comparisons employed Student's t-test (parametric) or Mann–Whitney U test (non-parametric). Categorical variables were reported as frequencies (percentages), with differences assessed using the Pearson's chi-squared test.

Univariable logistic regression identified recurrence-associated covariates. Results were presented as adjusted odds ratios (ORs) with 95% confidence intervals (CIs). The Benjamini–Hochberg procedure controlled false discovery rate (FDR) at q < 0.05, only FDR-significant variables advanced to multivariable logistic regression.

DFS curves were generated using the Kaplan–Meier method, with log-rank tests evaluating group differences. Variables significant (*P* < 0.05) in univariate analysis entered a Cox proportional hazards model for multivariable adjustment. Results were presented as adjusted hazard ratios (HRs) with 95% CIs.

To minimize indication bias in adjuvant chemotherapy analyses, we performed 1:1 propensity score matching (PSM) without replacement (caliper width: 0.02). Propensity scores were estimated using a multivariable logistic regression model (dependent variable: chemotherapy receipt; covariates: age, tumor size, FIGO stage, staging completeness, and omentectomy status). Nearest-neighbor matching ensured covariate balance, assessed via absolute standardized mean differences (SMD < 0.10 indicating adequate balance). Statistical significance was defined as two-tailed *P* < 0.05 throughout.

## Results

### Patients' characteristics and surgical management

A total of 338 cases of FIGO stage I AGCT were initially diagnosed between January 1988 and January 2024. After applying exclusion criteria, five patients were excluded due to loss to follow-up, yielding a final cohort of 333 eligible cases, which comprised 196 patients (58.9%) with FIGO stage IA and 137 patients (41.1%) with FIGO stage IC. Pathological upstaging was identified in six patients without grossly detectable extraovarian lesions who underwent comprehensive surgical staging. The findings included positive peritoneal washing cytology in three cases, with isolated instances of fallopian tube involvement (1 patient), omental metastasis (1 patient), and tumor detection in pelvic peritoneal biopsies (1 patient) observed during the procedures. Three cases were excluded from the analysis due to the inclusion criteria included only patients with stage I disease. No cases of FIGO stage IB were identified, as all patients presented with unilateral ovarian involvement. Table 1 summarized the baseline clinical characteristics stratified by FIGO stage.

**Table 1:** Clinical, surgical data, and oncological outcomes of patients with early-stage AGCT

Variables	Values			
	Total (*n* = 333)	IA (*n* = 196)	IC (*n* = 137)	*P*
Age (median)	48.1 ± 13.03	50 ± 12.812	45.38 ± 12.89	0.79
Tumor size (cm)				0.124
10	204 (61.3%)	124 (63.3%)	80 (58.4%)	
Exceeding 10	63 (18.9%)	31 (15.8%)	32 (23.4%)	
Unknown	66 (19.8%)	41 (20.9)	25 (18.2%)	
Symptoms at diagnosis				
Abnormal uterine bleeding	121 (36.3%)	81 (41.3%)	40 (29.2%)	
Asymptomatic	147 (44.1%)	88 (44.9%)	59 (43.1%)	
Abdominal distention with or without palpable adnexal mass	6 (1.8%)	2 (1.0%)	4 (2.9%)	
Abdominal pain	44 (13.2%)	18 (9.2%)	26 (19.0%)	
Unknown	15 (4.5%)	7 (3.6%)	8 (5.8%)	
Surgical approach				0.886
Laparoscopic	179 (53.8%)	106 (54.1%)	73 (45.9%)	
Transabdominal	154 (46.2%)	90 (53.3%)	64 (46.7%)	
Surgical procedure				0.422
FSS	130 (39.0%)	73 (37.2%)	57 (41.6%)	
RS	203 (61.0%)	123 (62.8%)	80 (58.4%)	
Staging surgery				0.006*
N	178 (53.5%)	117 (59.7%)	61 (44.5%)	
Y	155 (46.5%)	79 (40.3%)	76 (55.5%)	
Lymphadenectomy				0.962
N	228 (68.5%)	134 (68.4%)	94 (68.6%)	
Y	105 (31.5%)	62 (31.6%)	43 (31.4%)	
Omentectomy				0.013*
N	192 (57.7%)	124 (63.3%)	68 (49.6%)	
Y	141 (42.3%)	72 (36.7%)	69 (50.4%)	
Endometrial pathology				
Assessed	213 (64.0%)	124 (63.3%)	83 (60.6%)	
Normal endometrium	188 (88.3%)	108 (87.1%)	80 (96.4%)	
Hyperplasia with atypia	20 (9.4%)	18 (14.5%)	2 (2.4%)	
Carcinoma	5 (2.3%)	4 (3.2%)	1 (1.2%)	
Not assessed	120 (36%)	66 (33.7%)	54 (39.4%)	
Adjuvant chemotherapy				< 0.001*
N	225 (67.6%)	161 (82.1%)	64 (46.7%)	
Y	108 (32.4%)	35 (17.9%)	73 (53.3%)	
PEB	58 (53.7%)	16 (45.7%)	42 (57.5%)	
Others	50 (46.3%)	19 (54.3%)	31 (42.5%)	
Recurrence				< 0.001*
N	240 (72.1%)	158 (80.6%)	82 (59.9%)	
Y	93 (27.9%)	38 (19.4%)	55 (40.1%)	
Residual ovary	13 (14.0%)	5 (13.2%)	8 (14.5%)	
Pelvic	26 (28.0%)	11 (28.9%)	15 (27.3%)	
Abdominal	48 (51.6%)	18 (47.4%)	30 (54.5%)	
Distant	6 (6.4%)	4 (10.5%)	2 (3.6%)	
Death				0.1
N	326 (97.9%)	194 (99.0%)	132 (96.4%)	
Y	7 (2.1%)	2 (1.0%)	5 (3.6%)	
Recurrence Time (Month)		70.6 ± 48.5	70.1 ± 46.4	71.0 ± 50.4
Follow-up Time (Month)	138.5 ± 108.0	136.7 ± 106.0	141.0 ± 111.1	0.685

FSS, fertility-sparing surgery; RS, radical surgery; PEB, cisplatin, etoposide, bleomycin

No significant between-group differences were observed in age at diagnosis, median tumor size, surgical approach (open vs. minimally invasive), surgical procedure (FSS vs. RS), or lymphadenectomy rates. However, the stage IC group demonstrated a higher prevalence of elevated CA125 levels compared to stage IA counterparts (13.9% vs. 9.2%; *P* = 0.002). Significant disparities emerged in treatment patterns: patients with stage IC disease had higher comprehensive staging surgery rate (55.5% vs. 40.3%; *P* = 0.006), omentectomy rate (50.4% vs. 36.7%; *P* = 0.013), and adjuvant chemotherapy rate (52.6% vs. 18.4%; *P* < 0.001) (Table 1).

Among all early-stage patients, a total of 48 patients underwent secondary surgical resection of the residual adnexa (with or without comprehensive staging) following cystectomy, while 10 patients underwent only ovarian cystectomy and opted for follow-up observation. In both groups receiving adjuvant therapy, the PEB regimen (cisplatin, etoposide, bleomycin) constituted approximately 50% of first-line treatments. Carboplatin/paclitaxel (TC) emerged as the predominant alternative regimen in remaining cases (Table 1).

### Oncological outcomes

The median follow-up duration for the cohort was 117 months (range: 14–599). Among the 93 patients who developed recurrence, 38 cases (40.9%) were in the FIGO stage IA subgroup compared to 55 (59.1%) in stage IC (P < 0.001). The mean time to recurrence were comparable between two groups (71.0 ± 50.4 vs. 70.1 ± 46.4 months; P = 0.932). There was no statistically significant difference in recurrence rates between FSS and RS patients (P = 0.355). Notably, despite being early-stage cases, intra-abdominal recurrence occurred in nearly half of the patients across both FSS and RS groups (Table 1). Among FSS patients (n = 130), 90.0% of first recurrences (36/40) occurred within residual ovarian tissue or the pelvic-abdominal cavity, the remaining four developed liver parenchymal and full-thickness intestinal implantation metastasis at the first recurrence. Within the FSS cohort, recurrence rates differed by procedure: in the cystectomy-only subgroup (n = 10), 70% (7/10) recurred after a median follow-up of 52 months (range: 6–96), with 43% (3/7) at the ipsilateral adnexa. In contrast, the unilateral salpingo-oophorectomy (USO) subgroup (n = 120) had a recurrence rate of 27.5% (33/120) after a median follow-up of 67 months (range: 14–134). Among these recurrences, the patterns were primarily: pelvic-abdominal metastases (18/33, 54.5%), contralateral adnexal involvement (6/33, 18.2%) or isolated pelvic lesions (8/33, 24.2%).

Although with missing data for tumor size (19.8%) and CA125 (15.0%), sensitivity analysis using multiply imputed data for missing tumor size and CA125 confirmed the primary findings: neither tumor size nor CA125 level was associated with recurrence rates, DFS or OS (Table 2, 4 and Supplementary Table S2). Our analysis revealed significantly higher recurrence rates in patients at stage IC (40.1% vs 19.4%; FDR = 0.005), undergoing incomplete surgical staging (51.2% vs 34.4%; FDR = 0.016), without lymphadenectomy (35.8% vs 20.4%; FDR = 0.016) and omentectomy (46.7% vs 31.2%; FDR = 0.018), as well as those undergoing chemotherapy (49.5% vs 25.8%; FDR = 0.005). Furthermore, adjuvant therapy recipients demonstrated higher recurrence rates than observation counterparts (49.5% vs 25.8%; FDR < 0.001). However, analysis restricted to the stage IC subgroup alone revealed no factors associated with recurrence rate (Supplementary Table S1).

**Table 2:** Univariable analysis of recurrence risk factors with FDR correction

Factors	Total (*n* = 333)			
	NR	R	P	FDR
FIGO stage			< 0.001	0.005*
IA	158 (80.6%)	38 (19.4%)		
IC	82 (59.9%)	55 (40.1%)		
CA125 (U/ml)			0.359	0.462
Normal	205 (71.2%)	83 (28.8%)		
Elevated	35 (77.8%)	10 (22.2%)		
Tumor size			0.445	0.501
10 cm	187 (71.9%)	73 (28.1%)		
Exceeding 10 cm	53 (72.6%)	20 (27.4%)		
Surgical approach			0.998	0.998
Laparoscopic	129 (72.1%)	50 (27.9%)		
Transabdominal	111 (72.1%)	43 (27.9%)		
Surgical procedure			0.355	0.462
FSS	90 (69.2%)	40 (30.8%)		
RS	150 (73.9%)	53 (26.1%)		
Staging surgery			0.006	0.016*
N	117 (65.7%)	61 (34.3%)		
Y	123 (79.4%)	32 (20.6%)		
Lymphadenectomy			0.007	0.016*
N	154 (67.5%)	74 (32.5%)		
Y	86 (81.1%)	19 (17.9%)		
Omentectomy			0.01	0.018*
N	128 (66.7%)	64 (33.3%)		
Y	112 (79.4%)	29 (20.6%)		
Adjuvant chemotherapy			< 0.001	0.005*
N	178 (79.1%)	47 (20.9%)		
Y	62 (57.4%)	46 (42.6%)		

FIGO, International Federation of Gynecology and Obstetrics; FSS, fertility-sparing surgery; RS, radical surgery; NR, no recurrence; R, recurrence; FDR, False Discovery Rate

^*^Significant after FDR correction (q < 0.05)

Multivariable logistic regression analysis of the entire cohort identified stage IC, incomplete staging surgery, and adjuvant chemotherapy as independent factors associated with increased recurrence risk: stage IC (OR = 2.5, 95% CI 1.4–4.3; P = 0.001), incomplete staging (OR = 2.3, 95% CI 1.2–4.4; P = 0.015), and adjuvant chemotherapy (OR = 2.4, 95% CI 1.4–4.2; P = 0.002) (Table 3). In the stage IA subgroup specifically, only adjuvant chemotherapy was associated with a significantly higher recurrence risk (OR = 4.0, 95% CI 1.7–9.7; P = 0.002)..

**Table 3:** Multivariate logistic regression analysis in early-stage AGCT

Factors (*n* = 333)	OR	Cl	*p*-value
FIGO stage			
IA (Reference)			
IC	2.5	1.4–4.3	0.001
Staging surgery			
N	2.3	1.2–4.4	0.015
Y (Reference)			
Adjuvant Chemotherapy			
N (Reference)			
Y	2.4	1.4–4.2	0.002

FIGO, International Federation of Gynecology and Obstetrics; OR, Odds Ratio; Cl, confidence interval

**Fig. 1:**
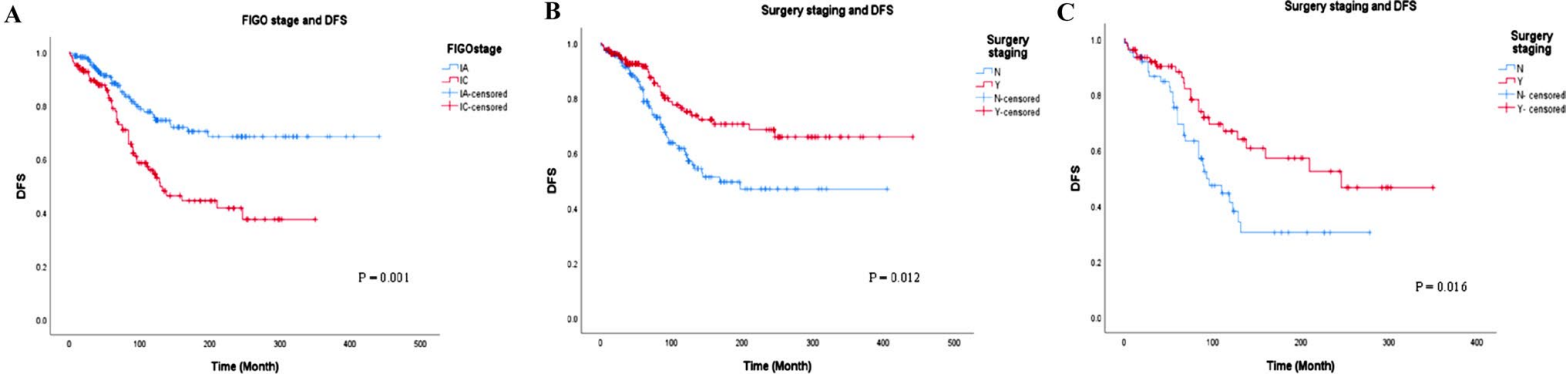
Factors associated with disease-free survival in the early-stage adult granulosa cell tumor. **A** FIGO stage associated with disease-free survival in the early-stage adult granulosa cell tumor; **B** Surgery staging associated with disease-free survival in the early-stage adult granulosa cell tumor; **C** FIGO stage associated with disease-free survival in the stage IC adult granulosa cell tumor

The overall mortality rate was 7.5% (7/93), with 2 deaths in stage IA (1.0%) and 5 in stage IC (3.6%) (*P* = 0.1). The 5-year DFS and OS rates for the entire cohort were 84.3% and 100%. Further analysis revealed that in patients with FIGO stage IA and IC, the 5-year DFS rates were 88.3% and 78.8% (*P* < 0.001) respectively, and both groups achieved 100% 5-year OS. Table 4 demonstrated risk factors for DFS in stage I AGCT patients. In univariate analysis, DFS showed significant correlations with initial FIGO staging (*P* = 0.005), surgery staging (*P* = 0.005), surgery approach (*P* = 0.028), lymphadenectomy (*P* = 0.004), omentectomy (*P* = 0.006) and adjuvant chemotherapy (*P* = 0.013). The level of CA125, tumor size and surgery type showed no significant association with DFS. The multivariable analysis demonstrated that FIGO IC disease and incomplete surgery staging were independent negative prognostic factors in DFS in patients with early-stage disease (HR = 2.2, 95% CI = 1.4–3.6, *P* = 0.001; HR = 2.7, 95% CI = 1.4–5.6, *P* = 0.012, respectively) (Fig. 1). However, no significant difference was observed when we analyzed the risk factors of OS in the whole cohort (Supplementary Table S2).

**Table 4:** Factors related to DFS in patients with early-stage AGCT

Factors		DFS					
		Univariate			Multivariate		
		HR	95% CI	*P*	HR	95% CI	*P*
Ovarian tumor size	10 cm (Ref)						
	> 10 cm	1.1	0.6–2.1	0.715			
CA125	Normal (Ref)						
	Elevated	1.2	0.6–2.2	0.893			
FIGO Stage	IA (Ref)						
	IC	2.3	1.5–3.4	< 0.001	1.2	1.4–3.6	0.001
Surgery type	FSS	1.4	0.9–2.1	0.111			
	RS (Ref)						
Surgery staging	N	1.9	1.2–2.9	0.005	2.7	1.4–5.6	0.012
	Y (Ref)						
Surgery approach	Laparoscopic	1.6	1.1–2.4	0.028			
	Transabdominal (Ref)						
Lymphadenectomy	Not performed	2.8	1.7–4.7	0.004			
	Performed (Ref)						
Omentectomy	Not performed	1.9	1.2–2.9	0.006			
	Performed (Ref)						
Adjuvant chemotherapy	N (Ref)						
	Y	1.7	1.1–2.5	0.013			

DFS, disease-free survival; FIGO, International Federation of Gynecology and Obstetrics; FSS, fertility-sparing surgery; RS, radical surgery; HR, Hazard Ratio; Cl, confidence interval

We further conducted a comprehensive analysis of high-risk factors associated with decreased DFS in both subgroups. Univariate analysis identified incomplete surgical staging, omission of lymphadenectomy, and lack of omentectomy as significant predictors of reduced DFS in both subgroups. However, multivariate analysis demonstrated that only incomplete surgical staging remained an independent prognostic factor for worse DFS in FIGO stage IC cases (HR = 2.5, 95% CI 0.4–14.2, *P* = 0.016), and none of these surgical parameters showed significant association with DFS outcomes in FIGO stage IA patients (Table 5).

**Table 5:** Factors related to DFS in patients with IA and IC AGCT

Factors		IC						IA		
		Univariate			Multivariate			Univariate		
		HR	95% CI	P	HR	95% CI	P	HR	95% CI	P
Surgery staging	N	2.0	1.1–3.4	0.002	2.5	0.4–14.2	0.016	2.5	0.9–3.2	0.004
	Y (Ref)									
Lymphadenectomy	Not performed	2.7	1.4–5.0	0.015				3.7	1.5–8.8	0.015
	Performed (Ref)									
Omentectomy	Not performed	1.8	1.0–3.0	0.045				3.1	1.4–7.0	0.017
	Performed (Ref)									

DFS, disease-free survival; FSS, fertility-sparing surgery; RS, radical surgery; HR, Hazard Ratio; Cl, confidence interval

### Propensity matched analysis

Following PMS (94 chemotherapy recipients vs. 94 non-recipients, total *n* = 188), the SMD values for FIGO stage were reduced to < 0.1. Balance improvements were also observed for age at diagnosis, tumor size, staging surgery, and omentectomy compared to the pre-matched cohort (Supplementary Table S3). In the matched cohort, adjuvant chemotherapy showed no significant association with recurrence risk (40.4% vs. 24.5%, OR = 1.9, 95% CI 1.0–3.7; *P* = 0.067) or DFS (HR = 1.3, 95% CI 0.8–2.2; P = 0.35) (Supplementary Table S4). Notably, in the matched stage IC subgroup, staging surgery remained significantly associated with DFS (HR = 4.0, 95% CI 1.5–10.3; *P* = 0.004), consistent with unmatched cohort results (Supplementary Table S5).

## Discussion

Approximately 80–90% of AGCT patients present with FIGO stage I disease [11]. Surgery remains the standard initial treatment for all AGCT patients, though postoperative adjuvant chemotherapy has evolved over the past decades [12, 13]. Despite advances in treatment and seemingly favorable clinical presentations, some patients diagnosed with early-stage AGCT experience recurrence and disease-related mortality [14–16]. As with other rare tumors, detailed information on the natural history and optimal treatment of AGCT patients is limited. Additionally, its indolent progression and the need for prolonged follow-up complicate efforts to understand biological behavior of the disease. Therefore, there is a need to more precisely define high-risk histopathological features in early-stage AGCT and establish optimal management strategies.

Current literature presents conflicting evidence regarding oncologic outcomes of RS for early-stage AGCTs. While some studies reported reduced recurrence rates and prolonged DFS associated with aggressive approaches [1, 17, 18], others demonstrated comparable recurrence and survival outcomes between radical and conservative procedures [6, 15, 19]. In our cohort of 130 FSS patients, recurrence rates showed no statistically significant difference compared to RS group. Furthermore, multivariate analysis revealed that FSS did not significantly compromise DFS. These findings strongly supported FSS as a viable oncological strategy for reproductive-age women with stage I AGCT, reflecting the tumor's inherent indolent biology and the curative potential of complete resection. Notably, cystectomy was associated with a 2.5-fold higher recurrence rate (70% vs. 27.5%; P < 0.01) and earlier median recurrence (52 vs. 67 months) compared to USO. This aligned with histopathological evidence of residual tumor in 16.3% of secondary surgeries, indicating that cystectomy’s failure to eliminate microscopic disease. Given that 43% of recurrences after cystectomy originated ipsilaterally, simple cystectomy cannot be recommended. USO—with thorough contralateral ovarian inspection—represents the optimal FSS approach, minimizing residual disease while preserving fertility. Comprehensive evaluation must include endometrial assessment, as concurrent endometrial pathology was identified in 10% of our cohort. Therefore, for early-stage patients undergoing FSS, USO is recommended alongside mandatory endometrial evaluation [20].

Our analysis identified completion of staging surgery demonstrated not only significantly reduced recurrence rates in the entire cohort (34.4% vs. 51.2%, *P* = 0.006) but also emerged as an independent predictor for improved DFS (*P* = 0.002). This protective association also remained in subgroup analyses for both stage IA (11.4% vs. 24.8%, *P* = 0.02) and IC (30.3% vs. 52.5%, *P* = 0.008) disease, with particularly notable DFS improvement observed in the IC subgroup through multivariate analysis (*P* = 0.016). This finding aligned with prior investigations emphasizing the critical role of comprehensive surgical staging in presumed early-stage disease [21–23]. Although comprehensive staging surgery might have no impact on the OS [24], we reckoned incomplete surgical staging still might pose potential clinical risks, including: failure to detect occult metastatic lesions, omission of indicated adjuvant chemotherapy in high-risk patients, and subsequent therapeutic inadequacy. Therefore, complete surgical staging, including peritoneal washing, inspection of peritoneal surfaces, random or oriented multiple biopsies, and omentectomy, is necessary in patients with presumed early-stage AGCT.

Current studies predominantly supported that staging procedures for AGCTs may include omentectomy but should not be extended to lymphadenectomy [7, 9, 25]. Although neither lymphadenectomy nor omentectomy was identified as an independent prognostic factor for better DFS in our overall early-stage cohort, we had two patients (2.0%) developed retroperitoneal lymph node metastases but 17 (12.1%) patients exhibited omentum metastases, indicating that lymphadenectomy should not be included in the routine surgical staging.

According to the NCCN guidelines, adjuvant chemotherapy is recommended for early-stage ovarian cancer patients exhibiting high-risk features, including tumor rupture, stage IC disease, poorly differentiated histology, or tumor size > 15 cm. However, a specific therapeutic consensus is lacking for stage I adult granulosa cell tumors (AGCTs) exhibiting high-risk characteristics. Previous studies demonstrated no significant improvement in DFS with adjuvant chemotherapy in FIGO stage IC AGCT patients [7, 8, 23]. In our cohort, initial multivariate analysis indicated higher recurrence risk with adjuvant chemotherapy (OR = 2.4, P = 0.002). This likely reflected indication bias, as chemotherapy was preferentially administered to high-risk patients (e.g., 40.1% of stage IC vs. 19.4% of stage IA patients). To address this, we performed PSM adjusted for age, FIGO stage, tumor size, staging completeness, and omentectomy status. Although higher recurrence rates were observed in the chemotherapy group, PSM confirmed no independent association between chemotherapy and recurrence or DFS, aligned with prior studies.

Consequently, we stratified stage IA and IC patients for further analysis. Univariate analyses within each stratum revealed no association between adjuvant chemotherapy and recurrence risk. Notably, residual indication bias may persist due to unmeasured confounders affecting chemotherapy decisions, despite PSM adjustment. Given the absence of demonstrable clinical benefit and potential treatment-related toxicities, we emphasized the critical need for multi-institutional studies to evaluate the efficacy of adjuvant therapy in early-stage AGCT and to establish the novel risk stratification to avoid unnecessary cytotoxic therapies [26].

To our knowledge, this study represents the largest retrospective analysis to date focusing on early-stage AGCT of the ovary, providing novel insights into surgical decision-making and postoperative surveillance strategies for this understudied population. We detailed the clinical characteristics and oncological outcomes of the entire early-stage cohort and revealed critical distinctions in recurrence patterns and key factors associated with DFS between stage IA and IC patients. Significantly, our findings demonstrated that although stage IC was categorized as early-stage disease, it exhibited a 2.5-fold increased recurrence risk and a sinificantly worse DFS rate compared to stage IA (HR = 2.2, *P* < 0.05). This underscored the necessity for comprehensive surgical staging, even when FSS was pursued. The study's longitudinal strength, with follow-up spanning 30 years, substantially enhanced detection of late recurrences and prognostic assessment, given the propensity of these tumors for recurrence beyond five years post-diagnosis.

However, as a retrospective, single-institution study, our findings might be subject to selection bias. Furthermore, due to the favorable prognosis of stage I AGCT (with few death events), the related factors of OS and more sensitive biomarkers for this tumor type are still need to be explored. Future multi-institutional, prospective studies utilizing pooled data and more rigorous statistical methods are warranted to further clarify prognostic factors, improve biomarker precision, and define optimal treatment strategies.

In conclusion, long-term surveillance is essential due to the risk of late recurrence. For reproductive-age women with stage I AGCT, FSS involving USO (not simple cystectomy) demonstrated oncologic safety, and concurrent endometrial evaluation is recommended. Complete surgical staging is critical to mitigate recurrence and improve DFS, particularly in stage IC, while lymphadenectomy appears unnecessary. After adjustment for indication bias via PSM, adjuvant chemotherapy showed no clear independent impact in early-stage AGCT. Prospective studies are needed to optimize management and prevent overtreatment.

## Author affiliations

Gynecology, Peking Union Medical College Hospital, Chinese Academy of Medical Sciences & Peking Union Medical College, Peking Union Medical College Hospital (Dongdan Campus), No.1 Shuaifuyuan Wangfujing Dongcheng District, Beijing, 100,730, China.

## Data availability statement

The datasets are not publicly available due to privacy reasons but are available from the corresponding author (caodongyan@pumch.cn) on reasonable request.

## Supplementary Information

Below is the link to the electronic supplementary material.

Supplementary file1 (DOCX 38 KB)

## Declarations

### Provenance and peer review

Not commissioned; externally peer reviewed.
