# Supplementary material for: Stage IA and IC adult granulosa cell tumors: Clinical features, long-term outcomes and prognostic factors in a 333-patient cohort over three decades
Source: Int J Clin Oncol. 2025 Oct 14;30(12):2643–51. doi: 10.1007/s10147-025-02897-w (PMC12644150; doi:10.1007/s10147-025-02897-w)
Supplement: Supplementary file 1 — Supplementary file1 (DOCX 38 KB) [file 10147_2025_2897_MOESM1_ESM.docx]

**Supplementary Table 1. Prognostic significances related to** **recurrence rate in FIGO stage IA and IC**

| **Factors** | IA (n = 196) | | |  |  | IC (n = 137) | | |  |
| --- | --- | --- | --- | --- | --- | --- | --- | --- | --- |
|  | NR | R | P | FDR |  | NR | R | P | FDR |
| **CA125 (U/ml)** |  |  |  |  |  |  |  |  |  |
| Normal | 140 (79.5%) | 36 (20.5%) | 0.262 | 0.419 |  | 65 (59.1%) | 45 (40.9%) | 0.713 | 0.914 |
| Elevated | 18 (90.0%) | 2 (10.0%) |  |  |  | 17 (63.0%) | 10 (37.0%) |  |  |
| **Tumor size** |  |  |  |  |  |  |  |  |  |
| 10 cm | 128 (80.0%) | 32 (20.0%) | 0.648 | 0.741 |  | 59 (59.0%) | 41 (41.0%) | 0.737 | 0.983 |
| exceeding 10 cm | 30 (83.3%) | 6 (16.7%) |  |  |  | 23 (62.2%) | 14 (37.8%) |  |  |
| **Surgical approach** |  |  |  |  |  |  |  |  |  |
| Laparoscopic | 85 (80.2%) | 21 (19.8%) | 0.871 | 0.871 |  | 44 (60.3%) | 29 (39.7%) | 0.915 | 0.989 |
| Transabdominal | 73 (81.1%) | 17 (18.9%) |  |  |  | 38 (59.4%) | 26 (40.6%) |  |  |
| **Surgical procedure** |  |  |  |  |  |  |  |  |  |
| FSS | 56 (76.7%) | 17 (23.3%) | 0.287 | 0.383 |  | 34 (59.6%) | 23 (40.4%) | 0.967 | 0.967 |
| RS | 102 (82.9%) | 21 (17.1%) |  |  |  | 48 (60%) | 32 (40%) |  |  |
| **Staging surgery** |  |  |  |  |  |  |  |  |  |
| N | 88 (75.2%) | 29 (24.8%) | 0.02 | 0.04* |  | 29 (47.5%) | 32 (52.5%) | 0.008 | 0.064 |
| Y | 70 (88.6%) | 9 (11.4%) |  |  |  | 53 (69.7%) | 23 (30.3%) |  |  |
| **Lymphadenectomy** |  |  |  |  |  |  |  |  |  |
| N | 102 (76.1%) | 32 (23.9%) | 0.019 | 0.0507 |  | 52 (55.3%) | 42 (44.7%) | 0.109 | 0.291 |
| Y | 56 (90.3%) | 6 (9.7%) |  |  |  | 30 (69.8%) | 13 (30.2%) |  |  |
| **Omentectomy** |  |  |  |  |  |  |  |  |  |
| N | 93 (75%) | 31 (25%) | 0.009 | 0.036* |  | 35 (51.5%) | 33 (48.5%) | 0.047 | 0.188 |
| Y | 65 (90.3%) | 7 (9.7%) |  |  |  | 47 (68.1%) | 22 (31.9%) |  |  |
| **Adjuvant chemotherapy** |  |  |  |  |  |  |  |  |  |
| N | 135 (84.4%) | 25 (15.6%) | 0.005 | 0.04* |  | 43 (66.2%) | 22 (33.8%) | 0.153 | 0.306 |
| Y | 23 (63.9%) | 13 (36.1%) |  |  |  | 39 (54.2%) | 33 (45.8%) |  |  |

FIGO: International Federation of Gynecology and Obstetrics; FSS: fertility-sparing surgery; RS: radical surgery; NR: no recurrence; R: recurrence; FDR: False Discovery Rate (Benjamini-Hochberg procedure).

* = Significant after FDR correction (q < 0.05)

**Supplementary Table 2. Factors related to OS in patients with early-stage AGCT**

| Factors (n = 333) | | OS |  |  |
| --- | --- | --- | --- | --- |
|  |  | Univariate | | |
|  |  | HR | 95% CI | P |
| Ovarian tumor size | 10 cm (Ref) |  |  |  |
|  | > 10 cm | 1.1 | 0.1–23.7 | 0.950 |
| CA125 | Normal (Ref) |  |  |  |
|  | Elevated | 0.3 | 0–50577.3 | 0.858 |
| FIGO Stage | IA (Ref) |  |  |  |
|  | IC | 3.8 | 0.7–19.6 | 0.113 |
| Surgery type | FSS | 1.0 | 0.2–5.4 | 0.968 |
|  | RS (Ref) |  |  |  |
| Surgery staging | N | 1.3 | 0.3–6.0 | 0.968 |
|  | Y (Ref) |  |  |  |
| Surgery approach | Laparoscopic | 1.4 | 0. 1–12.7 | 0.786 |
|  | Transabdominal (Ref) |  |  |  |
| Lymphadenectomy | Not performed | 0.1 | 0.1–2.5 | 0.442 |
|  | Performed (Ref) |  |  |  |
| Omentectomy | Not performed | 1.3 | 0.3–5.8 | 0.753 |
|  | Performed (Ref) |  |  |  |
| Adjuvant chemotherapy | N (Ref) |  |  |  |
|  | Y | 1.2 | 0.3–5.3 | 0.84 |

OS: overall survival; FIGO: International Federation of Gynecology and Obstetrics; FSS: fertility-sparing surgery; RS: radical surgery. HR: Hazard Ratio; Cl: confidence interval.

**Supplementary Table 3. Patient Demographics and Clinical Characteristics After Propensity Score Matching**

| **Variables** | NCT (n = 94) | CT (n = 94) | Pre-SMD | SMD | P | FDR |
| --- | --- | --- | --- | --- | --- | --- |
| **Age (Y)** |  |  | 0.359 | 0.281 | 0.056 | 0.126 |
| ≤ 50 | 27 (62.8%) | 16 (37.2%) |  |  |  |  |
| > 50 | 67 (46.2%) | 78 (53.8%) |  |  |  |  |
| **FIGO stage** |  |  | 0.307 | 0.022 | 0.880 | 0.880 |
| IA-B | 36 (50.7%) | 35 (49.3%) |  |  |  |  |
| IC | 58 (49.6%) | 59 (50.4%) |  |  |  |  |
| **Tumor size (cm)** |  |  | 0.240 | 0.129 | 0.491 | 0.552 |
| ≤ 10 | 70 (48.6%) | 75 (51.4%) |  |  |  |  |
| exceeding 10 | 24 (54.5%) | 20 (41.7%) |  |  |  |  |
| **CA125 (U/L)** |  |  | 0.232 | 0.128 | 0.382 | 0.573 |
| normal | 80 (48.8%) | 84 (51.2%) |  |  |  |  |
| elevated | 14 (58.3%) | 10 (36.4%) |  |  |  |  |
| **Surgery approach** |  |  | 0.016 | 0.214 | 0.144 | 0.324 |
| Laparoscopic | 54 (55.1%) | 44 (44.9%) |  |  |  |  |
| Transabdominal | 40 (44.4%) | 50 (55.6%) |  |  |  |  |
| **Surgical procedure** |  |  | 0.090 | 0.152 | 0.299 | 0.538 |
| FSS | 42 (54.5%) | 35 (45.5%) |  |  |  |  |
| RS | 52 (46.8%) | 59 (53.2%) |  |  |  |  |
| **Staging surgery** |  |  | 0.307 | 0.216 | 0.141 | 0.317 |
| N | 36 (43.9%) | 46 (56.1%) |  |  |  |  |
| Y | 58 (54.7%) | 48 (45.3%) |  |  |  |  |
| **Lymphadenectomy** |  |  | 0.105 | 0.245 | 0.418 | 0.537 |
| N | 54 (45.4%) | 65 (54.6%) |  |  |  |  |
| Y | 40 (58.0%) | 29 (42.0%) |  |  |  |  |
| **Omentectomy** |  |  | 0.106 | 0.137 | 0.106 | 0.238 |
| N | 36 (43.4%) | 47 (56.6%) |  |  |  |  |
| Y | 58 (55.2%) | 47 (44.8%) |  |  |  |  |

DFS: disease-free survival; FIGO: International Federation of Gynecology and Obstetrics; FSS: fertility-sparing surgery; RS: radical surgery; HR: Hazard Ratio; Cl: confidence interval.

**Supplementary Table 4: Matched Analysis of Recurrence rate and Cox Regression by the receipt adjuvant chemotherapy**

| **Group** | NCT | CT | P |
| --- | --- | --- | --- |
| **Recurrence rate** | 24.5% (23/94) | 40.4% (38/94) | 0.033 |
| OR (Cl) | Reference | 1.9 (1.0–3.7) | 0.067 |
| **5 years-DFS** | 70.5% | 60.0% | 0.346 |
| HR (Cl) | Reference | 1.3 (0.8–2.2) | 0.349 |

OR: Odds Ratio; HR: Hazard Ratio; Cl: confidence interval.

**Supplementary Table 5: Matched Analysis of DFS in FIGO IA and IC stage by the receipt adjuvant chemotherapy**

| Factors | | IC | | |  | IA | | | | |
| --- | --- | --- | --- | --- | --- | --- | --- | --- | --- | --- |
|  |  | Univariate | | |  | | Univariate | | |  |
|  |  | HR | 95% CI | P |  | | HR | 95% CI | P |  |
| Ovarian tumor size | 10 cm (Ref) |  |  |  |  | |  |  |  |  |
|  | > 10 cm | 0.7 | 0.4–1.5 | 0.414 |  | | 0.6 | 0.1–4.4 | 0.585 |  |
| CA125 | Normal (Ref) |  |  |  |  | |  |  |  |  |
|  | Elevated | 1.2 | 0.5–2.7 | 0.532 |  | | 0.04 | 0.0–26589.9 | 0.790 |  |
| Surgery type | FSS | 1.3 | 0.6–2.2 | 0.464 |  | |  |  |  |  |
|  | RS (Ref) |  |  |  |  | | 1.2 | 0.4–3.9 | 0.750 |  |
| **Surgery staging** | **N** | **2.0** | **1.1–3.7** | **0.001*** |  | |  |  |  |  |
|  | Y (Ref) |  |  |  |  | | 0.4 | 0.1–1.0 | 0.054 |  |
| Surgery approach | Laparoscopic | 1.5 | 0.8–2.6 | 0.216 |  | | 0.8 | 0.3–2.5 | 0.763 |  |
|  | Transabdominal (Ref) |  |  |  |  | |  |  |  |  |
| Lymphadenectomy | Not performed | 3.5 | 1.6–7.7 | 0.021 |  | | 2.8 | 1.0–8.3 | 0.068 |  |
|  | Performed (Ref) |  |  |  |  | |  |  |  |  |
| Omentectomy | Not performed | 1.9 | 1.0–3.6 | 0.031 |  | | 2.8 | 1.0–8.2 | 0.074 |  |
|  | Performed (Ref) |  |  |  |  | |  |  |  |  |
| Adjuvant chemotherapy | N (Ref) |  |  |  |  | |  |  |  |  |
|  | Y | 0.8 | 0.5–1.4 | 0.457 |  | | 2.0 | 1.0–13.9 | 0.054 |  |

DFS: disease-free survival; FIGO: International Federation of Gynecology and Obstetrics; FSS: fertility-sparing surgery; RS: radical surgery; HR: Hazard Ratio; Cl: confidence interval.

* = Significant after multivariate analysis (HR = 4.0, 95% CI 1.5–10.3; P = 0.004)
